# Wnt3a/β-Catenin/CBP Activation in the Progression of Cervical Intraepithelial Neoplasia

**DOI:** 10.3389/pore.2021.609620

**Published:** 2021-03-31

**Authors:** Dingqing Feng, Jie Lin, Wenhui Wang, Keqin Yan, Haiyan Liang, Jing Liang, Huan Yu, Bin Ling

**Affiliations:** ^1^ Department of Obstetrics and Gynecology, China-Japan Friendship Hospital, Beijing, China; ^2^ Department of Pathology, China-Japan Friendship Hospital, Beijing, China; ^3^ Chinese Academy of Medical Sciences and Peking Union Medical College, Beijing, China

**Keywords:** cervical intraepithelial neoplasia, wnt signaling, CBP, Piwil2, stemness, Differentiation

## Abstract

Piwil2 reprograms HPV-infected reserve cells in the cervix into tumor-initiated cells (TICs) and upregulates Wnt3a expression sequentially, which leads to cervical intraepithelial neoplasia (CIN) and ultimately squamous cell carcinoma (SCC). However, little is known regarding Wnt signaling in the maintenance of TIC stemness during the progression of cervical lesions. We herein investigated the expression of canonical Wnt3a signaling and related genes by microarray data set analysis and immunohistochemical (IHC) staining of samples obtained by biopsy of normal cervix, low- and high-grade CIN, and invasive SCC tissue. Array data analyzed by GEO2R showed higher expression levels of Wnt signaling and their target genes*,* significant upregulation of stemness-associated markers, and notably downregulated cell differentiation markers in CIN and SCC tissues compared with those in the normal cervix tissue. Further, Gene Set Enrichment Analysis (GSEA) revealed that Wnt pathway-related genes significantly enriched in SCC. IHC staining showed gradually increased immunoreactivity score of Wnt3a and CBP and notable translocation of *β*-catenin from the membrane to the cytoplasm and nucleus during the lesion progression. The intensity and proportion of P16, Ki67 and CK17 staining also increased with the progression of cervical lesions, whereas minimal to negative Involucrin expression was observed in CIN2/3 and SCC. Therefore, canonical Wnt signaling may contribute to the progression of CIN to SCC and may be a potential therapeutic target.

## Introduction

Cervical cancer is among the most preventable human malignancies, yet it remains a leading cause of death among women worldwide, accounting for more than 300,000 deaths annually [1]. In 2015, an estimated 98,900 new cases of invasive cervical cancer were diagnosed, and 30,500 women in China died from this cancer [2]. High-risk human papillomavirus (HR-HPV) is the major cause of cervical cancer, and in the precursor stages of cervical intraepithelial neoplasia (CIN, graded 1-3 according to severity), viral oncoprotein E6 and E7 targeting and degradation of P53 and retinoblastoma protein (pRb) lead to cell transformation, telomerase activation, and immortalization of the infected cells [3]. Further, it has been reported that E6 and E7 initiate the expression of Piwil2, which reactivates multiple germline factors of *c-Myc*, *Klf4*, *Sox2*, *Nanog*, and *Oct4* and reprograms HR-HPV-infected cells to tumor-initiating cells (TICs) [4]. The regulation of TIC stemness and differentiation plays a pivotal role in the progression of CIN to invasive cancer. However, the precise mechanism remains to be determined.

Wnts are secreted cysteine-rich glycoproteins that act as short-range ligands to locally activate receptor-mediated signaling pathways [5, 6]. A key step in the activation of Wnt target genes is the formation of a complex of *β*-catenin with members of the T-cell transcription factor (TCF)/lymphoid enhancer-binding factor (LEF) family of transcription factors [6–8]. To generate a transcriptionally active complex, TCF/β-catenin recruits the KAT3 transcriptional coactivator CREB-binding protein (CBP) or its closely related homolog, p300, as well as other components of the basal transcription machinery, to initiate transcription [6, 9, 10]. As Kahn and colleagues reported, CBP/β-catenin-mediated transcription is essential for stem and/or progenitor cell maintenance and proliferation, whereas a switch to p300/β-catenin-mediated transcription is a critical step in the initiation of differentiation and a decrease in cellular potency [6]. In addition to regulating cellular processes, including proliferation, differentiation, motility, and survival, the canonical *β*-catenin-dependent Wnt signaling pathway plays crucial roles in embryonic development and maintenance of homeostasis in mature tissues [11, 12]. Wnt signaling is also involved in stem cell maintenance by modulating the levels of intrinsic pluripotency factors, for example, Oct4, Nanog, and Sox2 [13–15], and Wnt3a supplementation has been demonstrated to maintain the pluripotency of embryonic stem cells [16, 17].

Accumulating evidence suggests that the Wnt/β-catenin signaling pathway is often involved in oncogenesis and cancer development [6, 11, 18]. The importance of aberrant Wnt signaling in some types of cancer, such as colorectal and breast cancer, is clear. In mice, it has long been known that misexpression of Wnt-1, -3, or -10 induces mammary adenocarcinomas [19, 20]. The APC^Min^ mouse has also been shown to exhibit an enhanced incidence (∼10%) of spontaneous mammary cancer and a greatly increased susceptibility (90%) to carcinogen-induced mammary cancer [21, 22]. However, aberrant Wnt signaling may play a role in many other types of malignancies, even those without the classical mutations associated with the pathway. Our previous study reported that the HPV16 oncoproteins E6 and E7 reprogrammed HaCaT cells and led to TIC formation by restoring Piwil2 expression [4]. We also observed that Piwil2 synchronously activated Wnt3a signaling to enhance reprogramming and maintain the stemness of TICs via the CBP/β-catenin interaction [data unpublished]. To explore the potential role of Wnt3a in the progression of cervical neoplasia, we investigated the expression of canonical Wnt3a signaling and related genes with microarray data set analysis and immunohistochemical staining of biopsied samples of normal cervix, low- and high-grade cervical intraepithelial neoplasia (CIN), and invasive cervical squamous cell carcinoma (SCC) tissue.

Our present findings provide novel pathological insights into Wnt3a signaling in the progression of CIN and aggressiveness of cervical cancer, thus defining a potential therapeutic target for cervical lesions.

## Materials and Methods

### Clinical Samples

The project was approved by the Ethics Committee of China-Japan Friendship Hospital (approval number: 2020–28-K20). One hundred thirty-four histological specimens, including hysterectomy tissues and loop/cone biopsied samples, were retrieved from the files of the Department of Pathology of China-Japan Friendship Hospital. The specimens consisted of normal cervix (*n* = 20), CIN1 (*n* = 36), CIN2/3 (*n* = 32), and invasive SCC (*n* = 46) tissues.

### Immunohistochemistry Analysis

Four-micrometer-thick formalin-fixed, paraffin-embedded tissue sections were deparaffinized with xylene and rehydrated through a graded series of ethanol. Antigen retrieval was performed with 0.01 M citrate buffer (pH 6.0) in a microwave oven for 10 min. After cooling to room temperature, the slides were incubated with 3% H_2_O_2_ for 10 min to inactivate endogenous peroxidase. The slices were incubated overnight at 4°C in a humidified chamber with the following primary antibodies: 1:500 diluted anti-Wnt3a (ab28472), 1:500 diluted anti-β-catenin (ab32572), 1:1000 diluted anti-CBP (ab50702), 1:100 diluted anti-cytokeratin 17 (CK 17, ab53707), 1:1000 diluted anti-involucrin (ab53112), 1:200 diluted anti-CDKN2A/p16INK4α (p16, ab54210), and 1:200 diluted anti-Ki67 (ab16667). All of the primary antibodies were purchased from Abcam, United States. The binding of the primary antibodies was visualized using the ChemMate detection kit (PV-9000, ZSBIO, China). The slices were lightly counterstained with Mayer’s hematoxylin for 30 s.

Immunoreactivity was semiquantitatively evaluated. The degree of positive staining for all antibodies was evaluated by score on a scale of 0–3 for strength of intensity of staining and on a scale of 0–4 for percentage of positive cells. The final total score was generated by the intensity score × proportion score. The intensity score was defined as follows: 0, no staining of epithelial cells; 1, weak staining; 2, moderate staining; or 3, strong staining. The proportion score was defined as follows: 0, no staining of the cervical epithelial cells in any field; 1, <10% of the epithelium stain-positive; 2, 11–50% stain-positive; 3, 51–80% stain-positive; or 4, >80% stain-positive. The total score ranged from 0 to 12. Two different pathologists evaluated all the specimens in a blinded manner. Notably, the distribution of staining in the intraepithelial lesions (CIN1 and CIN2/3) was compared with that of the normal cervical squamous epithelium. CIN tissues were categorized as having the thickness of the samples being from two-thirds to fully stained.

### Microarray and Gene Set Enrichment Analysis

To investigate the expression pattern of genes associated with the activation of Wnt/β-catenin signaling in the progression of cervical lesions, the data set GSE64217 (containing 2 normal cervix, 2 CIN2/3, and 2 cervical squamous cell cancer samples), GSE51993 (containing 7 HPV- normal cervix samples, 9 HPV + CIN I samples and 8 HPV + CIN III samples), GSE67522 (containing 11 HPV16- normal cervix samples, 11 HPV16+ non-malignants and 20 HPV16 + cervical cancer tissues) were downloaded from the GEO database (https://www.ncbi.nlm.nih.gov/geo/). The platforms for these data sets are all based on GPL10558 and an Illumina HumanHT-12 V4.0 Expression BeadChip. The downloaded files were processed using the R package and then calibrated, standardized, and converted to log2 data, or normalized with cubic spline method. The limma package was used to standardize the gene chip and analyze the differential expression. The quantile normalized values for canonical Wnt signaling pathway associated genes were found out via *VLOOKUP* from the gene expression description tables. Boxplot was used to show the expression difference among groups of normal cervix, CIN and SCC.

### Statistical Analyses

The data are presented as the means ± SD. Statistical analyses were performed with SPSS version 25 (SPSS Inc., Chicago, IL, United States). The significance of gene expression between groups in microarray data sets were analyzed with one-way analysis of variance. The comparison of immunoreactivity score between normal cervix and cervical lesions were performed with two-tailed Chi-square test. Pearson test was used to analyze the correlation between the expression of the biomarkers and the cervical lesion degree. A *p* value <0.05 was considered significant.

## Results

### Expression Pattern of Canonical Wnt Pathway-Related Genes

**FIGURE 1:**
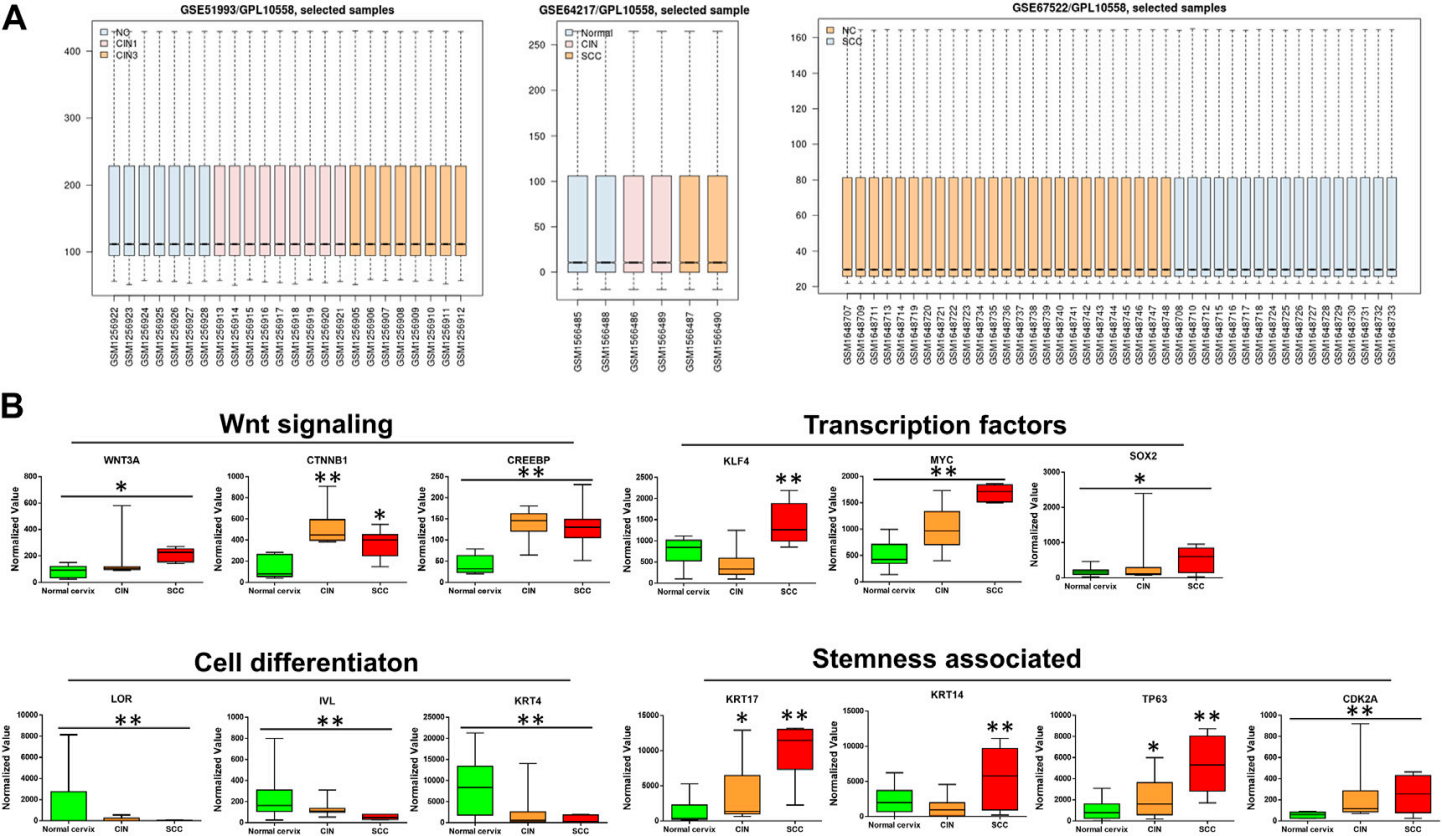
The bioinformatics analyses of the microarray data sets of GSE64217, GSE51993 and GSE67522 containing normal cervix, CIN, and SCC sample information **(A)** Boxplot of the distribution of normalized expression values **(B)** The microarray data sets of GSE64217, GSE51993 and GSE67522 were analyzed by GEO2R on GEO database. *Wnt3a/CTNNB1/CREBBP* and their target genes *MYC* and *SOX2* showed higher expression levels in CIN and SCC than in normal cervix. The stemness-associated markers were significantly upregulated, while the cell differentiation markers were notably downregulated in CIN and SCC. The expression values of KLF4 and CK14 decreased moderately in CIN as compared with normal cervix. CIN and SCC compared with normal cervix, *p < 0.05, **p < 0.01.

To identify the differentially expressed genes (DEGs), the data set GSE64217, GSE51993 and GSE67522 were analyzed by the GEO2R method on the GEO website. The boxplot of the distribution of the normalized expression values for selected samples demonstrates that the data were suitable for the analysis and “limma” (Figure 1A), which was used to perform a top table computation to extract a list of the top-ranked genes. Within the top 250 differentially expressed genes, the canonical Wnt signaling genes *Wnt3a*, *CTNNB1 (β-catenin)*, and *CREBBP (CBP)* were significantly upregulated in the samples of precancerous and cancer of the cervix compared with those of the normal cervix, thereby contributing to the distinct upregulation of their downstream transcription factors *KLF4*, *MYC*, and *SOX2*, except *KLF4* slightly downregulated in CIN (Figure 1B). As previously reported, CBP/β-catenin signaling is important for the maintenance of stem cell pluripotency [6]. *KRT17 (CK17)*, *TP63* and *CDK2A (P16)*, markers of HPV target cells/cervical cancer stem cells (CSCs), were apparently upregulated in CIN and SCC. In contrast, markers of squamous cell differentiation, *LORICRIN*, *INVOLUCRIN* and *KRT4 (CK4),* were significantly downregulated in CIN and SCC (Figure 1B). It was somewhat unusual that *KRT14 (CK14)* and *KLF4,* showed in these GSE gene sets, were moderately downregulated in CIN tissues compared with normal cervix, nevertheless they were all significantly upregulated in SCC tissues (Figure 1B).

### Wnt3a/β-Catenin/CBP Expression and Cervical Lesion Grade

**FIGURE 2:**
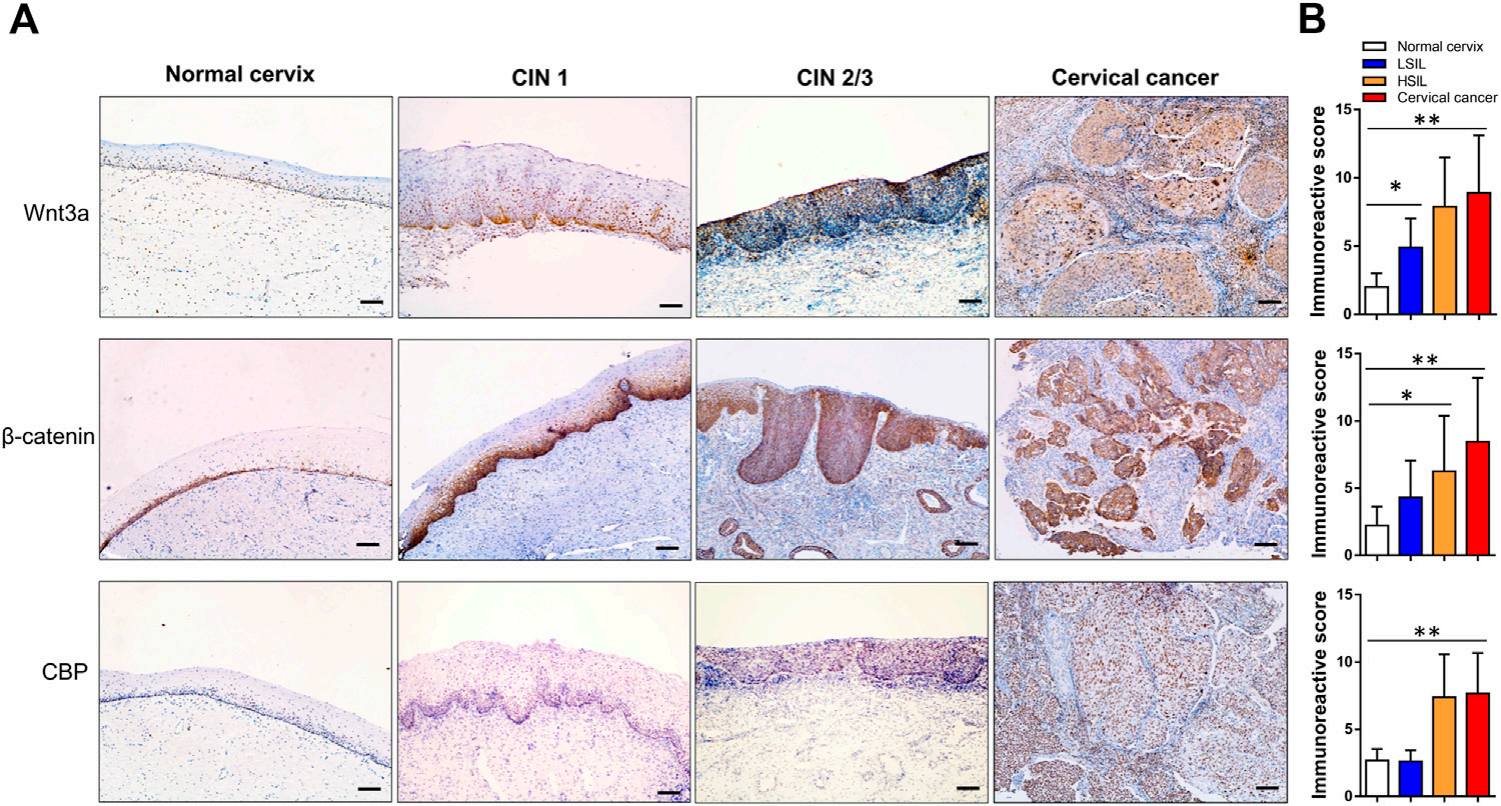
Immunohistochemical expression pattern of Wnt3a, *β*-catenin and CBP in normal cervix, CIN1, CIN2/3, and SCC tissue. In the normal cervix, Wnt3a-, *β*-catenin- and CBP-positive cells scattering was confined to the basal cell layer. The immunoreactivity score of Wnt3a and CBP gradually increased, and *β*-catenin staining showed a notable positive translocation from the membrane to the cytoplasm and nucleus during the progression from CIN1 to SCC **(A)**. The total scores of Wnt3a, *β*-catenin and CBP immunoreactivity were significantly higher in CIN2/3 and cervical cancer tissues compared to the normal cervix and CIN1 tissues, respectively **(B)**. *p < 0.05, **p < 0.01; Bar, 100 μm.

In normal cervical squamous epithelium, Wnt3a staining was confined to the basal cells, whereas in CIN1 diffuse immunoreactivity was limited to the lower one-third of the epithelial layer, and in CIN2/3, the staining extended through the full thickness of the epithelial layer (Figure 2A). Strong to moderate Wnt3a immunoreactivity was observed in 45 (97.8%) of 46 invasive SCCs and 26 (81.3%) of 32 CIN2/3, while 44.4% (16/36) CIN1 showed light-to-moderate expression, as occasional scattered staining was observed within basal cells in normal cervix that was considered negative (Table 1).

**TABLE 1:** Relationship between Wnt3a/β-catenin/CBP and lesion grade of cervical squamous epithelium (n=134).

Pathologic diagnosis	*N*	Wnt3a			β-catenin			CBP		
		Positive^a^ (%)	*χ* ^2^	*p*	Positive^a^ (%)	*χ* ^2^	*p*	Positive^a^ (%)	*χ* ^2^	*p*
Normal cervix	20	0	69.263^b^	<0.01^b^	1 (5%)	40.504^b^	<0.01^b^	2 (10%)	21.484^b^	<0.01^b^
CIN1	36	16 (44.4%)	32.438^c^	<0.01^c^	18 (50%)	11.735^c^	<0.01^c^	18 (50%)	7.195^c^	<0.05^c^
CIN2/3	32	26 (81.3%)			23 (71.9%)			20 (62.5%)		
Cervical cancer	46	45 (97.8%)			39 (84.8%)			36 (78.3%)		

^a^ Positive staining surpassed the basal cells.

^b^ Chi square test among groups of normal cervix, CIN1, CIN2/3, and cervical cancer.

^c^ Chi square test among lesion groups.

A gradual increase in the *β*-catenin score and its positive translocation from the membrane to the cytoplasm and finally to the nucleus was noted in cervical lesions at higher stages (Figure 2A). Only 5% (1/20) of the normal cervix samples were membrane positive for *β*-catenin in the parabasal cells, while one-half (18/36) of the CIN1 samples were membrane positive for *β*-catenin. Approximately 71.9% (23/32) of the CIN2/3 and 84.8% (39/46) of the SCC samples had cytoplasmic and even nuclear positive staining. The pattern of CBP expression in each stage of cervical lesion was similar to that of *β*-catenin with only nuclear immunoreactivity (Table 1). Overall, the total scores of Wnt3a, *β*-catenin and CBP immunoreactivity were significantly higher in CIN2/3 and cervical cancer tissues compared to the normal cervix and CIN1 tissues, respectively (Figure 2B, *p* < 0.01).

### Wnt Signaling Activation Promotes Lesion Progression

**FIGURE 3:**
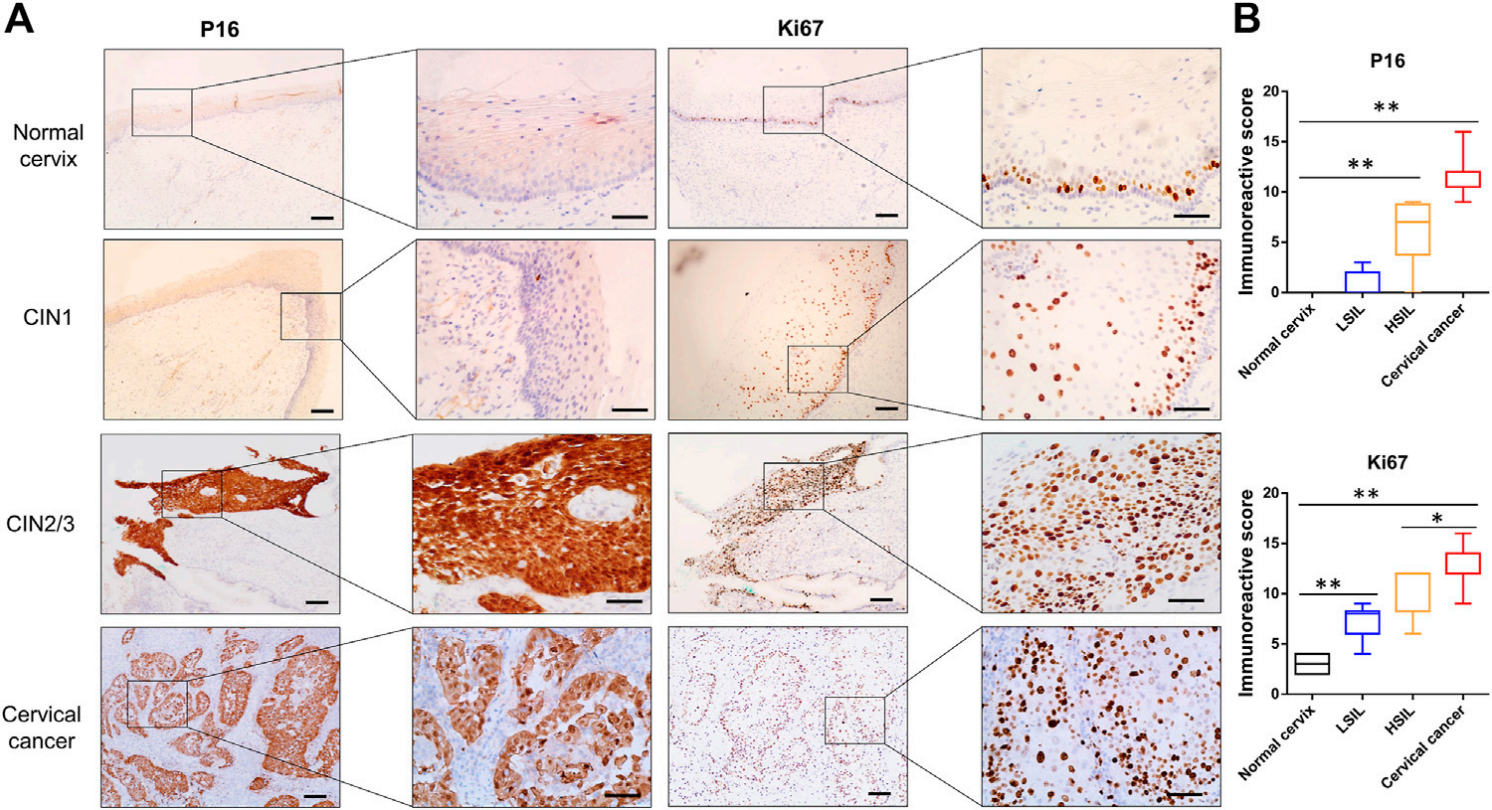
Representative images of P16 and Ki67 immunostaining in normal cervix, CIN1, CIN2/3, and SCC tissue **(A)** P16 was negative in the normal cervix and CIN1 samples. Scattered Ki67 positivity was confined to the basal cells in the normal cervix but in both the basal and parabasal cells in the CIN1 samples. Diffuse immunoreactivity of P16 and Ki67 was distributed in the SCC and full thickness of the CIN2/3 samples **(B)** The IHC scores of P16 and Ki67 were significantly higher in both the CIN2/3 and SCC tissues compared to the CIN1 and normal cervix. *p < 0.05, **p < 0.01; Bar, 100 μm.

Markers useful in the assessment of lesion grade were further observed. P16, which is valuable in the diagnosis of high-grade CIN, was positively stained in the upper two-thirds and full thickness of the epithelium in most cases of CIN2/3 and in all of cervical cancer tissues, while minimal or negative P16 was observed in cervical squamous epithelium of the normal cervix or CIN1 samples (Figure 3A). As the Ki67 antigen is expressed in all active phases of the cell cycle, diffuse immunoreactivity was distributed throughout SCC and the full thickness of the CIN2/3 samples, while scattered positive staining was confined to only the lower one-third of the CIN1 and the basal cells in the normal cervix samples (Figure 3A). Statistical analysis showed that the IHC scores of P16 and Ki67 were significantly higher in both the CIN2/3 and SCC tissues compared to the CIN1 and normal cervix (Figure 3B, *p* < 0.01).

**FIGURE 4:**
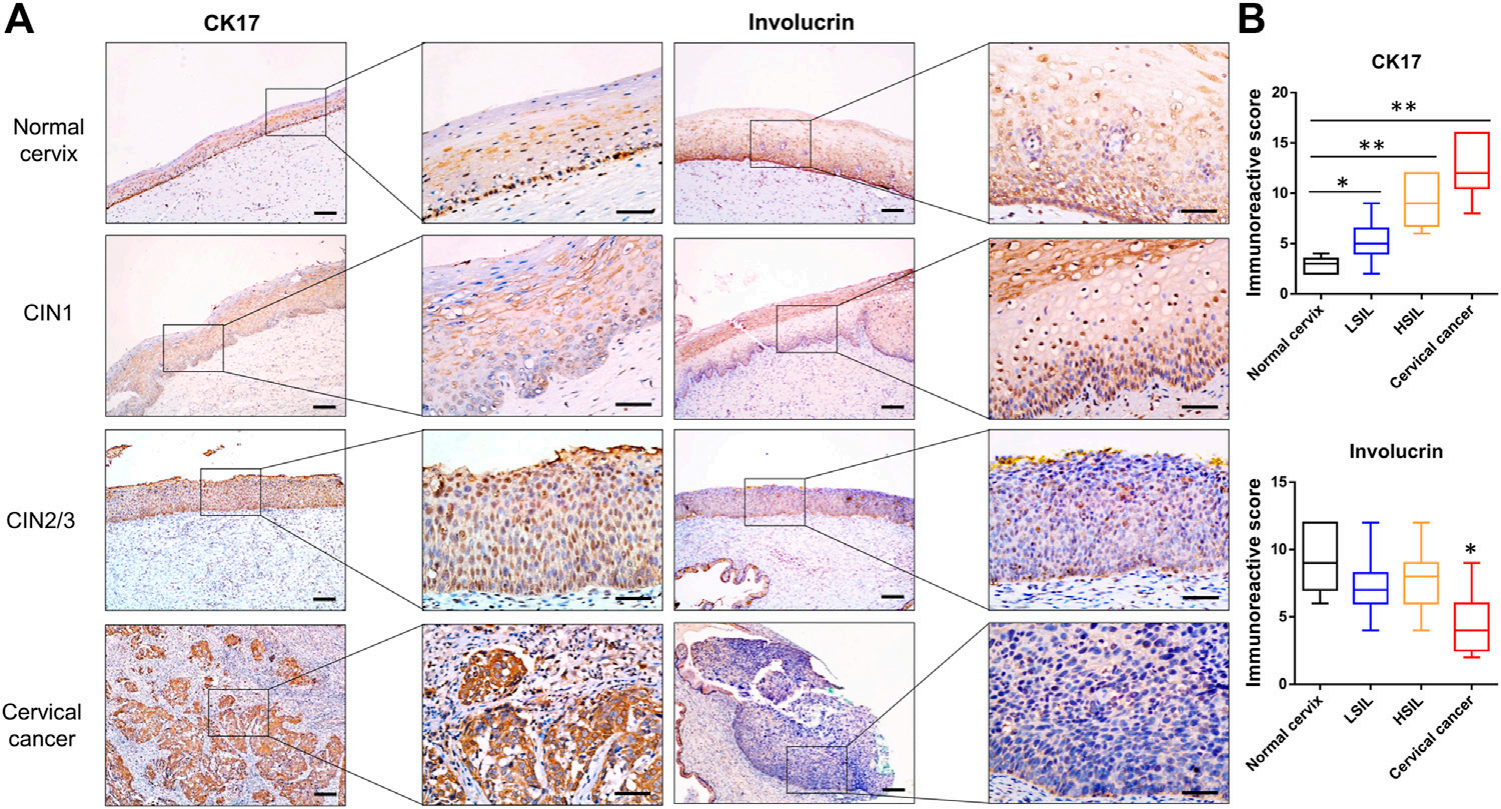
Representative images of CK17 and Involucrin immunostaining in normal cervix, CIN1, CIN2/3, and SCC tissues **(A)** The reserve cells in the basal layer were positive for CK17 in the normal cervix. The full thickness of the CIN2/3 and SCC samples showed CK17 staining, whereas only a very small number of positive cells were scattered in the lower one-third of the squamous epithelium in the CIN1 samples. In contrast to the pattern of CK17 expression, diffuse immunoreactivity of Involucrin was observed in the full thickness of the normal cervix and CIN1 samples, whereas minimal and negative Involucrin expression was observed in the CIN2/3 and SCC tissue **(B)** The IHC score of CK17 increased gradually in accord with the lesion grade, while the score of Involucrin staining was significantly lower in SCC than that in normal cervix and CIN lesions. *p < 0.05, **p < 0.01; Bar, 100 μm.

In this work, the characterization of *β*-catenin and coactivator CBP expression implied that the canonical Wnt signaling pathway was activated in high-grade lesions. Consistent with previous studies, CBP-Wnt activity was associated with tumorigenesis and stem cell maintenance because it modulates the levels of intrinsic pluripotency factors, such as Oct4, Nanog, and Sox2 [6]. Here, CK17, a marker of reserve cells and TICs [23, 24], was expressed in the basal compartment of the normal cervix and CIN1 samples, while it displayed diffuse immunoreactivity in the SCC and CIN2/3 samples (Figure 4). On the other hand, the differentiation marker Involucrin was extensively expressed in the upper two-thirds of the squamous epithelium in the normal cervix and CIN1 samples, while minimal immunoreactivity was observed in the CIN2/3 or SCC samples (Figure 4).

### Correlation Between the Expression of Various Biomarkers With the Cervical Lesion Grade

**FIGURE 5:**
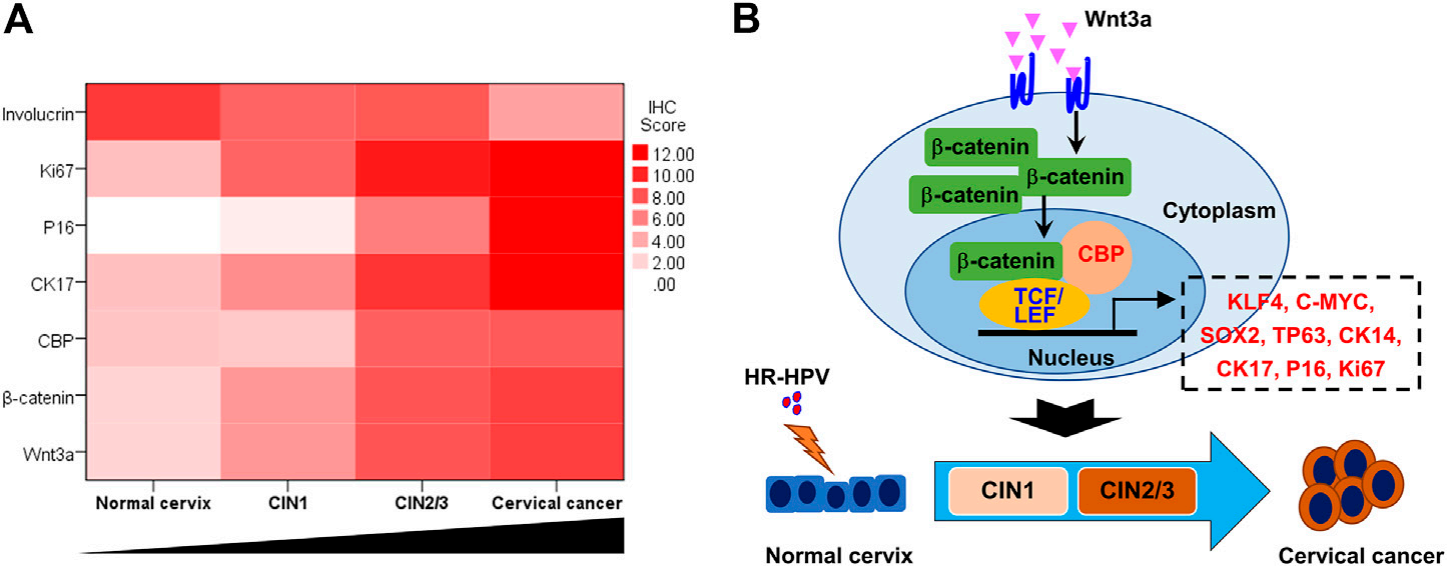
Wnt signaling pathway activation and cervical lesion progression **(A)** The heat map, which was made with the total IHC scores of various biomarkers and the cervical lesion grade, apparently showed the trends of Wnt/β-catenin/CBP-regulated gene expression in the process of cervical lesion **(B)** Model of Wnt3a activation promoting the transition from HR-HPV-infected cervix to cervical cancer.

The heatmap, which was made with the total IHC scores of various biomarkers and the cervical lesion grade, apparently displayed the association between them (Figure 5A). Spearman test further verified that the expression of Wnt3a, *β*-catenin, CBP, P16, Ki67, and CK17 were positively correlated with the cervical lesion grade (*r* = 0.679, 0.577, 0.688, 0.898, 0.892, 0.867, respectively, all *p* value <0.01), while the expression of Involucrin was negatively correlated with it (*r* = −0.557, *p* < 0.01) (Table 2). Taken together, these data suggest that Wnt/β-catenin/CBP is activated in CIN2/3 lesions and SCC, where it maintains TIC pluripotency and promotes cell proliferation, thereby contributing to neoplastic development.

**TABLE 2:** Correlation between the expression of various biomarkers and the cervical lesion grade.

	Total immunoreactivity scores						
	Wnt3a	β-catenin	CBP	P16	Ki67	CK17	Involucrin
Cervical lesion grade							
Correlation coefficient	0.679	0.577	0.688	0.898	0.892	0.867	−0.557
*p* Value	<0.01	<0.01	<0.01	<0.01	<0.01	<0.01	<0.01

## Discussion

In this report, we described the identification of canonical Wnt signaling activation in precancerous lesions and cervical cancer. Wnt proteins comprise a large family of secreted growth factors that play essential roles in regulating cell proliferation, survival, differentiation, tissue architecture, and organogenesis during embryonic development [6–9]. Accumulating evidence suggests that the Wnt/β-catenin signaling pathway serves critical functions in the sequential development of the neoplastic process from initiation, proliferation and transformation in a broad range of cancer types, such as colorectal, breast, and cervical cancer [7, 11, 18, 24–27]. The activation of the canonical Wnt pathway is characterized by the accumulation of *β*-catenin in the cytoplasm and nucleus; nevertheless, *β*-catenin is mainly located at cellular junctions in the absence of Wnt ligands [28–30].

In this study, the gene expression profile data showed that within the top 250 differentially expressed genes, the canonical Wnt signaling genes *Wnt3a*, *β-catenin*, and *CBP* were significantly upregulated in the CIN lesion and cervical cancer samples compared with those in the normal cervix sample. These bioinformatics data were further verified by immunohistochemical staining. In particular, a gradual increase in the *β*-catenin immunoreactivity score and its positive translocation from the membrane to the cytoplasm and finally to the nucleus were observed in CIN2/3 and SCC, which indicates the activation of Wnt signaling [6, 8, 12]. These excess *β*-catenin translocated to the nucleus and activated the downstream genes expression, including c-Myc, Sox2, Nanog, Oct4, Survivin and Cyclin D1, thus contributing to oncogenesis and cancer development [6, 27, 31].

The physical association between *β*-catenin and CBP/p300, which are cotranscriptional factors, influences Wnt/β-catenin signaling [6, 10, 32]. CBP-mediated Wnt activity is essential for cell proliferation and pluripotency and has a central role in advanced stages of neoplastic development [6, 33], whereas p300/β-catenin-mediated transcription is the critical step in initiating differentiation and decreasing cellular potency [10, 32, 34]. The activation of CBP/β-catenin is deemed the default active pathway in stem cells, which maintains an undifferentiated proliferative state [35, 36]. For regular development to proceed, cells must exit the cell cycle and initiate the process of differentiation [37–39]. The array data analysis performed with GEO2R showed that the expression of *CBP/β-catenin* and its target genes of *KLF4*, *MYC*, and *SOX2* were upregulated, generally synchronicity with the progression of cervical lesion grade. Consistent with the activation of CBP/β-catenin, markers of TICs *CK17*, *CK14*, *TP63* and *P16* were upregulated, while markers of squamous cell differentiation *LORICRIN*, *INVOLUCRIN* and *CK4* were downregulated significantly in the CIN and SCC samples compared with normal cervix samples. However, the expression of *KLF4* and *CK14* were slightly decreased in CIN as compared with normal cervix. We speculated that the lesion grade (CIN1 or CIN2/3) and the status of high-risk HPV infection in CIN samples, to some extent, may interfere the results. The immunoreactivity scores analyzed with Spearman test showed that the expression of Ki67, P16, CK17 were positively correlated with the cervical lesion degree while Involucrin was just the opposite. Thus, both array and IHC data further verified that canonical Wnt signaling activation promotes cervical lesion progression, which indicates the potential role of Wnt3a in maintaining stemness of TICs reprogrammed by Piwil2 in our previous report and sequentially triggering the progression of CIN2/3 and SCC [4]. Furthermore, inhibiting the CBP/β-catenin interaction with specific antagonists (i.e., ICG-001 or PRI-724) led to symmetric CSC divisions at the expense of asymmetric divisions; thus, CSCs are stochastically cleared from their niche via symmetric differentiative division [40–42]. Therefore, selectively antagonizing the CBP/β-catenin interaction may be a potential target for CIN2/3 and SCC therapy.

In conclusion, our observations demonstrate that the canonical Wnt signaling pathway was activated during the oncogenesis of cervical lesions. The CBP/β-catenin interaction played an essential role in the progression of CIN and SCC by maintaining the stemness of TICs (Figure 5B). Therefore, targeting the CBP/β-catenin interaction may be a promising therapeutic strategy for preventing CIN progression.

## Data Availability

The datasets presented in this study can be found in online repositories. The names of the repository/repositories and accession number(s) can be found in the article/Supplementary Material.
